# Effect of 2-week postpartum check-ups on screening positive for postpartum depression: a population-based cohort study using instrumental variable estimation in Japan

**DOI:** 10.1093/fampra/cmad074

**Published:** 2023-07-19

**Authors:** Naoko Nakamura, Toshiharu Mitsuhashi, Yasuko Nakashima, Naomi Matsumoto, Takashi Yorifuji

**Affiliations:** Department of Epidemiology, Okayama University Graduate School of Medicine, Dentistry and Pharmaceutical Sciences, Okayama, Japan; Center for Innovative Clinical Medicine, Okayama University Hospital, Okayama, Japan; Health Promotion Division, Tsuyama City Department of Children’s Health, Tsuyama, Japan; Department of Epidemiology, Okayama University Graduate School of Medicine, Dentistry and Pharmaceutical Sciences, Okayama, Japan; Department of Epidemiology, Okayama University Graduate School of Medicine, Dentistry and Pharmaceutical Sciences, Okayama, Japan

**Keywords:** community health planning, diagnostic screening programmes, home visits, postpartum depression, postnatal care, quasi-experimental study

## Abstract

**Background:**

Postpartum depression is experienced by approximately 10% of women and affects the health and development of their children. Although it is recommended that all mothers have the opportunity for early detection and intervention for postpartum depression, it is unclear whether early postpartum check-ups help to reduce postpartum depression.

**Objective:**

The aim of this study was to assess the effect of 2-week postpartum check-ups on screening positive for postpartum depression in Japan.

**Methods:**

This was a population-based cohort study that used the administrative database of Tsuyama, Japan. Participants were women who received postpartum home visits from a public health nurse in Tsuyama during the fiscal years 2017–2019. Data were obtained on participant’s attendance at a 2-week postpartum check-up and their responses on the Edinburgh Postpartum Depression Scale. Owing to the initiation of a publicly funded postpartum check-up programme, participants were pseudo-randomly assigned to receive/not receive a 2-week postpartum check-up. We conducted instrumental variable estimation to assess the causal effects of the check-up on screening positive for postpartum depression.

**Results:**

The characteristics of the 1,382 participants did not differ by fiscal year of childbirth. We found a 6.7% (95% confidence interval 2.2–11.2) reduction in the prevalence of screening positive for postpartum depression as an effect of 2-week postpartum check-ups among women received 1-month postpartum home visits.

**Conclusion:**

The results suggest that 2-week postpartum check-ups are effective in reducing the prevalence of screening positive for postpartum depression among 1-month postpartum women. Despite some limitations, early postpartum care could reduce postpartum depression.

**Key messages**

- Two-week postpartum check-ups are effective in reducing EPDS-positives.
- The effect is shown in 1-month postpartum, when healthcare can be fragmented.
- Early postpartum check-ups are important for reducing postpartum depression.

## Background

Postpartum depression is defined in clinical practice and research as minor or major depression experienced by women in the first postpartum year^1,2^ and has a prevalence of approximately 7–13%.^2^ Substantial evidence shows that postpartum depression has adverse effects on externalizing behaviours and socioemotional development in children by increasing the risk of interparental conflict and mother–infant attachment disorder.^3^ In addition, untreated maternal depression can compromise the infant’s health more directly; for example, by leading to child abuse, neglect, and a failure to implement injury prevention for the child.^1^

To enable early postpartum depression detection and intervention, the main international guidelines recommend that all perinatal women should be screened for depression.^4–6^ Regular contact with healthcare providers in the early postpartum period, and thus the opportunity for screening, is desirable.^6^ However, it has been argued that postpartum routine check-ups of 4–6 weeks, which is a traditional practice in Western countries, are too late.^7,8^ Currently, the World Health Organization recommends that all mothers have contact with healthcare providers on postpartum days 3 and 7–14, and at 6 weeks,^9^ and the American College of Obstetricians and Gynecologists recommends that all women receive their first opportunity for postpartum care within 3 weeks after delivery.^10^

In Japan, all mothers traditionally receive an obstetrician check-up at 1-month postpartum and a home visit within 4 months postpartum from public health nurses or other childcare support staff. However, a recent study indicated that the prevalence of postpartum depression in Japan is highest (approximately 14%) in the first month after childbirth.^11^ To provide earlier postpartum support, in 2017, the Japanese government established a programme to subsidize the cost of two check-ups in the postpartum period. This policy has gradually led to the implementation of 2-week postpartum check-ups in addition to the traditional 1-month postpartum check-ups at the autonomous community level. However, few studies have examined the association between early postpartum check-ups and postpartum depression, and to the best of our knowledge, there are no population-based studies. Therefore, we evaluated the effect of 2-week postpartum check-ups on the prevalence of screening positive for postpartum depression using population-based data.

## Methods

### Design and setting

This was a population-based cohort study using administrative databases held by the city of Tsuyama, Okayama, Japan. Tsuyama is located in a hilly and mountainous area and has a population of approximately 100,000. A publicly funded 2-week postpartum check-up began in Tsuyama at the beginning of the fiscal year (FY) 2019 (i.e. April 2019). Prior to this, there had been no opportunity for routine visits (including self-funded check-ups) before 1-month postpartum. This resulted in a situation where women who gave birth in FY 2019 were pseudo-randomly assigned to receive a 2-week postpartum check-up. We used this quasi-experimental context to conduct instrumental variable (IV) estimation to assess the causal effects of a 2-week postpartum check-up on screening positive for postpartum depression.

### Data source

We used 2 kinds of databases from the Tsuyama maternal and child health services. One database contained information about delivery and postpartum conditions collected at postpartum home visits by public health nurses, who referred to birth records and conducted a questionnaire and interview with each mother. The other database included individual-level information on the use of the postpartum check-up tickets issued by municipal offices for reserving publicly funded check-ups. In these databases, each mother has a unique identifier; therefore, individual records are linked across databases and remain anonymous.

### Participants

**Fig. 1.**
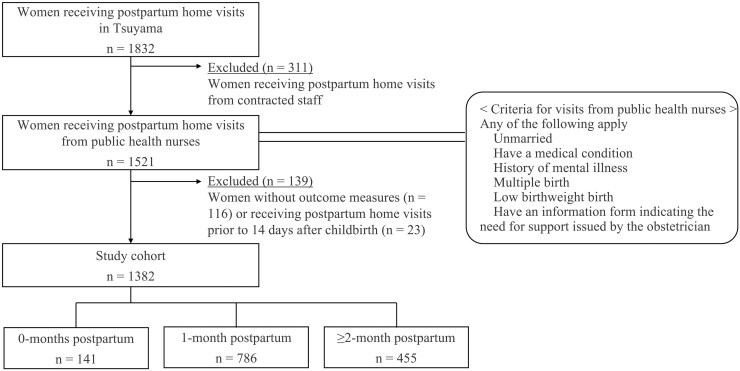
Cohort flow diagram showing the number of study participants receiving postpartum home visits in Tsuyama, Japan (2017–2019), including stratification by visit time and criteria for visits from public health nurses.

The source population was all postpartum women residing in Tsuyama during the FYs 2017–2019 (i.e. 2017 April 1 to 2020 March 31) who were eligible for the postpartum home visiting programme. We restricted our analysis to women who received a postpartum home visit during the study period from a public health nurse trained in postpartum depression screening. Women eligible for such visits were those who fitted any of several criteria (shown in Fig. 1) that indicated a risk of depression or child abuse, or the need for special care. We excluded women with missing outcome measure data and those who received a home visit prior to 14 days postpartum, which may have been before the 2-week postpartum check-up (Fig. 1). The study period ended in FY 2019 because of concerns about the effect of the coronavirus pandemic in Japan.

We stratified participants into 3 subgroups according to the timing of their postpartum home visit: 0 months postpartum (14 days to < 1 month postpartum), 1-month postpartum (1 month to < 2 months postpartum), and ≥ 2 months postpartum, for the following reason. First, each participant was assessed for postpartum depression at the home visit; however, the point prevalence of postpartum depression generally depends on which point in the postpartum period it is measured. Second, participants in each subgroup may have had a different risk level of postpartum depression because participants with preexisting mental illness (prevalence unknown) or at higher risk for such illness were visited earlier by public health nurses.

### Measures

#### Exposure variable

 The exposure of interest was attendance at a publicly funded 2-week postpartum check-up. This was measured by the first use of postpartum check-up tickets, because the 2 issued tickets were typically used at 2 weeks and 1 month postnatally, respectively. However, if a woman used a ticket only once after 28 days postpartum, it was defined as attendance at a 1-month (not 2-week) postpartum check-up.

In the subanalysis, the exposure was defined as childbirth in the FY 2019, to assess the effect of implementing the publicly funded postpartum check-up programme (i.e. the intention-to-treat effect, in contrast to the main analysis to assess the effect of attendance).

#### Instrumental variable

**Fig. 2.**
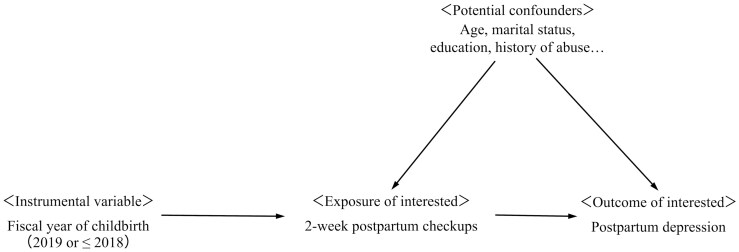
Directed acyclic graph showing the structure of instrumental variable analyses for evaluating the effect of postpartum check-ups on postpartum depression.

Women who receive postpartum check-ups and those who do not are likely to have different sociodemographic characteristics. A previous study found that the risk factors for failure to attend postpartum check-ups included younger age, unmarried, lower education, lower household income, smoking habits, and history of abuse,^12^ which may be common causes of postpartum depression. To take into account unmeasured confounding by these and other factors, the present study used IV estimation. IV can provide a simulation of the random assignment of exposure variables to participants. In this study, women who gave birth in FY 2019 were eligible for publicly funded 2-week postpartum check-ups, so childbirth in FY 2019 or prior to this year was defined as a binary IV (Fig. 2).

#### Outcome variable

Screening positive was defined as a score above the threshold of 8/9 on the Edinburgh Postpartum Depression Scale (EPDS), which was completed by participants at the postpartum home visits. The EPDS consists of 10 self-reported questions; total scores range from 0 to 30. The scale was developed by Cox et al.^13^ in 1987 and has been translated into multiple languages. It is the most widely used postpartum depression screening tool in primary care. Okano created a Japanese version of the EPDS and validated it with 1-month postpartum women; the scale showed a sensitivity and specificity of 75% and 93%, respectively, using a threshold of 8/9.^14^ This cutoff value has been frequently used in clinical and research fields in Japan and was therefore used in this study.

### Statistical analysis

We conducted a 2-stage least-squares (2SLS) estimation using IV and reported the estimates with 95% confidence intervals (CIs) to evaluate the effect of 2-week postpartum check-ups on screening positive. Although a linear model does not constrain the probability of a binary outcome to between 0 and 1, 2SLS estimates are close to those of methods that take such constraints into account.^15^ An estimated 2SLS coefficient represents the “compliers” average causal effect (CACE) of the exposure. Here, “compliers” are individuals who actually expose when assigned to an exposed group and do not actually expose when assigned to a nonexposed group.

For the sensitivity analysis, we performed regression adjustment. This method estimates the average causal effect in the whole population under the assumption that a sufficient set of confounding for adjustment has been measured,^16^ unlike the IV method, which estimates the CACE even under the presence of unobserved confounding if certain conditions are met. Missing covariate values were eliminated by listwise deletion. We used a linear model in the subanalysis to evaluate the intention-to-treat effect.

All statistical analyses were conducted using STATA version 17.0 (StataCorp LLC, College Station, TX, USA).

## Results

### Description of the participants

Of the 1,832 women who received postpartum home visits in Tsuyama during FYs 2017–2019, 1,382 were eligible for study inclusion (Fig. 1). Participants accounted for 60.0% of the 2,304 births in Tsuyama during the same period,^17^ although the exact proportion was unknown because the postpartum visit period differed slightly from the childbirth period.

Table 1 shows the characteristics of the participants by FY of childbirth in each of the 3 subgroups, stratified by the timing of the home visits. The characteristics did not differ by FY, except for some differences in child birthweight, the obstetrical institutions where women gave birth, and smoking habits (however, smoking habits had many missing values).

**Table 1.** Clinical and demographic characteristics in each visit time subgroup of 1,382 postpartum women receiving home visits in Tsuyama, Japan (2017–2019), by fiscal year of childbirth.

	0-month postpartum group		1-month postpartum group		≥2-month postpartum group	
	≤FY 2018	FY 2019	≤FY 2018	FY 2019	≤FY 2018	FY 2019
	*n* = 112	*n* = 29	*n* = 582	*n* = 204	*n* = 332	*n* = 123
Days postpartum at the time of home visits, median (IQR)	23 (19–26)	24 (20–26)	47 (41–54)	48 (41–55)	75 (66–92)	75 (68–85)
Age						
Years, mean (SD)	30.9 (6.4)	31.3 (6.2)	30.4 (5.3)	31.2 (5.3)	31.4 (5.4)	31.0 (4.9)
Missing, *n* (%)	0 (0.0)	1 (34.5)	1 (0.2)	2 (1.0)	1 (0.3)	0 (0.0)
Child’s birth weight, *n* (%)						
<1,500 g	1 (0.9)	0 (0.0)	1 (0.2)	1 (0.5)	8 (2.4)	2 (1.6)
1,500–2,499 g	16 (14.3)	2 (6.9)	49 (8.4)	22 (10.8)	48 (14.5)	4 (3.3)
2,500–3,999 g	90 (80.4)	27 (93.1)	524 (90.0)	179 (87.8)	272 (81.9)	116 (94.3)
≤4,000 g	3 (2.7)	0 (0.0)	5 (0.9)	2 (1.0)	2 (0.6)	1 (0.8)
Missing	2 (1.8)	0 (0.0)	3 (0.5)	0 (0.0)	2 (0.6)	0 (0.0)
Parity, *n* (%)						
Primipara	57 (50.9)	15 (51.7)	265 (45.5)	86 (42.2)	163 (49.1)	54 (43.9)
Missing	3 (2.7)	0 (0.0)	5 (0.9)	0 (0.0)	2 (0.6)	0 (0.0)
Smoking habit, *n* (%)						
Yes	3 (2.7)	4 (13.8)	16 (2.8)	3 (1.5)	10 (3.0)	1 (0.8)
Missing	26 (23.2)	10 (34.5)	108 (18.6)	70 (34.3)	63 (19.0)	41 (33.3)
Obstetrical institutions, *n* (%)						
A	41 (36.6)	10 (34.5)	70 (12.0)	26 (12.8)	49 (14.8)	17 (13.8)
B	29 (25.9)	9 (31.0)	209 (35.9)	69 (33.8)	79 (23.8)	43 (35.0)
C	23 (20.5)	5 (17.2)	126 (21.7)	45 (22.1)	59 (17.8)	21 (17.1)
D	11 (9.8)	3 (10.3)	81 (13.9)	21 (10.3)	34 (10.2)	16 (13.0)
Out-of-city	3 (2.7)	1 (3.5)	72 (12.4)	22 (10.8)	87 (26.2)	20 (16.3)
Missing	5 (4.5)	1 (3.5)	24 (4.1)	21 (10.3)	24 (7.2)	6 (4.9)
Feeding methods, *n* (%)						
Breastfeeding	17 (15.2)	6 (20.7)	208 (35.7)	77 (37.8)	122 (36.8)	55 (44.7)
Combination	80 (71.4)	20 (69.0)	311 (53.4)	102 (50.0)	140 (42.2)	52 (42.3)
Formula feeding	12 (10.7)	3 (10.3)	59 (10.1)	21 (10.3)	63 (19.0)	15 (12.2)
Missing	3 (2.7)	0 (0.0)	4 (0.7)	4 (2.0)	7 (2.1)	1 (0.8)
Breastfeeding problems, *n* (%)						
Yes	18 (16.1)	3 (10.3)	60 (10.3)	18 (8.8)	29 (8.7)	7 (5.7)
Missing	5 (4.5)	1 (3.5)	21 (3.6)	5 (2.5)	4 (1.2)	1 (0.8)

FY, fiscal year; IQR, interquartile range; SD, standard deviation.

### Description of postpartum check-ups and EPDS scores

Table 2 shows descriptive data for 2-week postpartum check-up attendance and EPDS assessment. In women who gave birth in FY 2019, when the publicly funded check-up system was launched, approximately 80% received 2-week postpartum check-ups, and the proportions were similar for all subgroups. For women who gave birth in FY 2018 or before, the attendance proportion was 0% in all subgroups because publicly funded check-ups did not exist at that time. As a reference for the proportion of attendance at postpartum check-ups in Japan, in FY 2019, 87.6% of infants received check-ups at 1 month of age^18^; these were usually conducted at the same time as the mothers’ check-ups. In our study, the attendance proportion at 1-month postpartum check-ups in FY 2019 was 94.7%, slightly higher than the national average.

**Table 2.** Descriptive statistics for 2-week postpartum check-up attendance and indicator of postpartum depression in each visit time subgroup of 1,382 postpartum women receiving home visits in Tsuyama, Japan (2017–2019), by fiscal year of childbirth.

	0-month postpartum group		1-month postpartum group		≥2-month postpartum group	
	≤FY 2018	FY 2019	≤FY 2018	FY 2019	≤FY 2018	FY 2019
	*n* = 112	*n* = 29	*n* = 582	*n* = 204	*n* = 332	*n* = 123
2-week postpartum check-ups						
Attendance, *n* (%)	0 (0.0)	23 (79.3)	0 (0.0)	164 (80.4)	0 (0.0)	96 (78.1)
Days postpartum, median (IQR)	-	13 (12–14)	-	13 (12–15)	-	13 (12–15)
Percentage of attendees underwent EPDS assessment, *n* (%)	-	23 (100)	-	164 (100)	-	96 (100)
EPDS assessment at home visits						
EPDS-positives, *n* (%)	20 (17.9)	4 (13.8)	40 (6.9)	3 (1.5)	18 (5.4)	2 (1.6)

EPDS, Edinburgh Postpartum Depression Scale; FY, fiscal year; IQR, interquartile range; SD, standard deviation.

The 2-week postpartum check-ups were implemented approximately 13 days after delivery, with little variation among all subgroups, and always included EPDS assessment.

The results of the EPDS assessment at home visits showed that a proportion of the positives (score of ≥ 9) in the 0-month postpartum group were more than twice as high as in the other subgroups.

### Main findings

Table 3 shows the results of the analysis for each subgroup. We found a 6.7% (95% CI 2.2–11.2) reduction in the prevalence of screening positive for postpartum depression in the 1-month postpartum group as an effect of 2-week postpartum check-ups. However, there was no significant reduction in the 0-month or ≥ 2-month postpartum group, according to the 2SLS estimation using IV. The result of the regression adjustment analysis was similar; moreover, the subanalysis results were consistent, although the estimates were somewhat smaller.

**Table 3.** Effect of 2-week postpartum check-up on screening positive for postpartum depression in each visit time subgroup of 1,382 postpartum women receiving home visits in Tsuyama, Japan (2017–2019).

Effect of check-ups	Two-stage least squares ^a^			Regression adjustment ^b^		
	*n*	The difference in prevalence, % (95% CI)		*n*	The difference in prevalence, % (95% CI)	
0-month postpartum group	141	−5.1 (−24.5 to 14.3)		98	−4.0 (−17.4 to 9.4)	
1-month postpartum group	786	−6.7 (−11.2 to −2.2)		567	−5.8 (−8.5 to −3.1)	
≥2-month postpartum group	455	−4.9 (−10.3 to 0.6)		321	0.7 (−5.2 to 6.6)	
Effect of programme	Linear regression					
	*n*	The difference in prevalence, % (95% CI)				
0-month postpartum group	141	−4.1 (−19.6 to 11.5)				
1-month postpartum group	786	−5.4 (−9.0 to -1.8)				
≥2-month postpartum group	455	−3.8 (−8.0 to 0.5)				

CI, confidence interval.

^a^The *F*-statistics from the first stage are 423.2, 2,380.1, and 1,175.3 from the top row, respectively.

^b^Adjusted for age, parity, smoking habit, obstetrical institution, feeding methods, and breastfeeding problems.

## Discussion

### Interpretation

The results of this study using population-based data from a small regional city in Japan showed that a 2-week postpartum check-up was effective in reducing the prevalence of EPDS positives by 6.7% for women who received postpartum home visits in the first month after childbirth. The estimated effect of postpartum check-ups may include the effect of postpartum depression screening because the check-up is an important screening opportunity (and indeed EPDS assessment was always carried out at the check-ups in this study). One systematic review^19^ showed that postpartum depression screening programmes may reduce the prevalence of postpartum depression by 4.7–9.1%; our results are consistent with this estimate. In this review, half of the trials included participants with known depression at baseline, as in the present study. However, an effect of the screening programmes was suggested even for those participants, albeit in a sense more akin to management than early detection. Such an effect may have been observed in our study because the postpartum check-ups also included the continuation of support and referral to specialists according to the screening results.

Our finding that the significant effect was restricted to the 1-month postpartum group can be interpreted in two ways. The first is that the effect of 2-week postpartum check-ups may be noticeable from 1 month to < 2 months after childbirth. This is notable given that the point prevalence of postpartum depression in Japan is reportedly greatest then.^11^ During this period, mothers often feel uncertain about who to consult about their postnatal concerns owing to the separation of maternal care and paediatric care^10^; this is also the case in Japan. The reduction in the prevalence of EPDS-positives during this high-risk period may have been because postpartum check-ups play an important role as an opportunity to connect all mothers to healthcare in the earlier postpartum period.

The second interpretation is that the effect of 2-week postpartum check-ups may be apparent for the subgroup at moderate risk of depression because the 1-month postpartum group may have had a risk level intermediate between the 0-month and ≥ 2-month postpartum group in this study. This supports the concept of a population approach, which assumes that the effect of a population-wide intervention is more prominent in majorities at the average risk level. Such approaches have been criticized for their lack of benefit to socially vulnerable groups^20^; however, this assumption could not be adequately assessed in this study. To assess this, studies are needed that focus on high-risk groups; namely, women who have received home visits at 0-month postpartum or who have declined them.

In this study, similar results were obtained from the IV estimation and regression adjustment, which suggested that the causal effect on the compliers was equivalent to that on the whole population. This indicates that the findings can be generalized to a population represented by approximately 60% of all postnatal women in Tsuyama, the study population. However, they do not include women who declined a postpartum home visit or who did not meet the criteria for public health nurse visits; therefore, the generalizability of the findings may be limited.

The subanalysis results showed that the effect of the publicly funded postpartum check-up programme was comparable to the effect of check-up attendance. The findings may partly represent the effects of the programme on non-participants of postpartum check-ups owing to growing public attention to postpartum depression.

### Strengths

Using IV analysis, we were able to compare the different exposure groups with theoretically no unmeasured confounding, such as by history of mental illness and social factors. We used the FY of childbirth in the context of the initiation of the new policy implementation as the IV, which was considered to have met the following 4 conditions necessary for an IV.^16^ First, an IV assigns the exposure with high probability. This was demonstrated to be met in this study by the high attendance proportion at 2-week postpartum check-ups, and the *F*-statistic (Table 3). Second, an IV does not directly affect the outcome other than through its effect on the exposure. This condition was considered to have been met within our study period, owing to the exclusion of FY 2020, which probably had a direct association with postpartum depression because of the effect of the coronavirus epidemic in Japan. Third, an IV has no common cause with the outcome. No events occurred in Japan around FY 2019 that would have affected the distribution of the characteristics of postpartum women, as shown by the data in Table 1. Fourth, no “defiers” exist. In this study, it was unlikely that anyone would have attended the check-ups if they were ineligible for the publicly funded check-up programme; similarly, it was unlikely that anyone would not have attended if they were eligible.

The other strength of our study was the low probability of exposure misclassification by measuring the usage of the check-up tickets. Moreover, it is unlikely that many nonexposed women received interventions with effects similar to those of the exposure. This point was mentioned as an issue in two previous randomized controlled trials evaluating the effect of early postpartum check-ups.^21,22^ In both these studies, many participants had contacted a doctor in the early postpartum period separately from check-ups. This was because a high proportion of the participants were willing to see a doctor or had health problems, resulting in similar interventions for both groups; therefore, no effect of the check-up was found. However, our participants were a more general population of postpartum women than in previous studies. Additionally, no facilities in Tsuyama provided self-funded 2-week postpartum check-ups; thus, there would have been few visits other than the publicly funded 2-week postpartum check-ups.

Furthermore, in our study, the EPDS was always administered at the postpartum check-ups, which may have minimized variation in the quality of the exposure. This contrasts with a previous UK report that more than 80% of general practitioners had not used screening tools, including the EPDS, during routine consultations with perinatal women.^23^ Because of the clear policy directive about postpartum check-ups in Japan, all exposed women in this study underwent almost homogeneous screenings.

### Limitations

This study could not assess changes over time in the prevalence of postpartum depression. Therefore, it is possible that the observed effect of the 2-week postpartum check-up also included a declining trend of EPDS-positives over time.

Second, we used the EPDS as a proxy for postpartum depression, and such self-reported screening tools reportedly estimate a higher prevalence of postpartum depression than diagnosis by physician interviews.^24^ Furthermore, the validity of the EPDS thresholds may differ depending on the timing of measurement,^25^ which was not considered in this study. Nonetheless, any outcome misclassification would have been nondifferential for the groups compared in this study; therefore, the result would be biased toward the null.

Finally, as mentioned in the methods section, the subgroups had different risk levels for postpartum depression, as well as different timing of postpartum screening, which somewhat complicated the interpretation. To address this problem, the whole population should be followed up at the same time, or stratified arbitrarily using the same criteria.

## Conclusion

In this population-based cohort study, a 2-week postpartum check-up was shown to be effective in reducing the prevalence of EPDS-positives at 1-month postpartum by approximately 7%. Although we recognize the need for a careful appraisal of generalizability, it seems evident that providing early postpartum women with opportunities for healthcare, including depression screening, could help to reduce postpartum depression.

## Data Availability

The data underlying this article cannot be shared publicly due to the fact that they contain information that could compromise the privacy of the study participants.
